# Supplementary material for: Deep serological profiling of the Trypanosoma cruzi TSSA antigen reveals different epitopes and modes of recognition by Chagas disease patients
Source: PLoS Negl Trop Dis. 2023 Aug 9;17(8):e0011542. doi: 10.1371/journal.pntd.0011542 (PMC10441789; doi:10.1371/journal.pntd.0011542)
Supplement: S1 Table — (DOCX) [file pntd.0011542.s001.docx]

**Supplementary Table 1: Information about the population study analyzed in this work.**

| **Sample ID** | **Sample Name** | **Sample Code** | **Chagas Serology** | **Sex** | **Age*** | **Region Code** | **Region** | **Contact** |
| --- | --- | --- | --- | --- | --- | --- | --- | --- |
| 01-0013-4 | 43508 | AR_P1 | POSITIVE | NA | 44 | AR | Argentina | Jaime Altcheh |
| 01-0014-3 | 43532 | AR_P2 | POSITIVE | NA | 31 | AR | Argentina | Jaime Altcheh |
| 01-0015-2 | 43539 | AR_P3 | POSITIVE | NA | 29 | AR | Argentina | Jaime Altcheh |
| 01-0016-1 | 43541 | AR_P4 | POSITIVE | NA | 21 | AR | Argentina | Jaime Altcheh |
| 01-0017-0 | 43560 | AR_P5 | POSITIVE | NA | 31 | AR | Argentina | Jaime Altcheh |
| 01-0018-0 | 43672 | AR_P6 | POSITIVE | NA | 33 | AR | Argentina | Jaime Altcheh |
| 01-0019-9 | 43857 | AR_N1 | NEGATIVE | NA | 39 | AR | Argentina | Jaime Altcheh |
| 01-0020-4 | 43925 | AR_N2 | NEGATIVE | NA | 15 | AR | Argentina | Jaime Altcheh |
| 01-0021-3 | 44003 | AR_N3 | NEGATIVE | NA | 27 | AR | Argentina | Jaime Altcheh |
| 01-0022-2 | 44005 | AR_N4 | NEGATIVE | NA | 14 | AR | Argentina | Jaime Altcheh |
| 01-0023-1 | 44059 | AR_N5 | NEGATIVE | NA | 28 | AR | Argentina | Jaime Altcheh |
| 01-0024-0 | 44067 | AR_N6 | NEGATIVE | NA | 19 | AR | Argentina | Jaime Altcheh |
| 01-0025-0 | 871 | AR_E1 | POSITIVE | NA | 66 | AR | Argentina | Jaime Altcheh |
| 01-0026-9 | 891 | AR_E2 | POSITIVE | NA | 54 | AR | Argentina | Jaime Altcheh |
| 01-0027-8 | 896 | AR_E3 | POSITIVE | NA | 43 | AR | Argentina | Jaime Altcheh |
| 01-0028-7 | 897 | AR_E4 | POSITIVE | NA | 44 | AR | Argentina | Jaime Altcheh |
| 01-0029-6 | 898 | AR_E5 | POSITIVE | NA | 56 | AR | Argentina | Jaime Altcheh |
| 01-0030-1 | 899 | AR_E6 | POSITIVE | NA | 43 | AR | Argentina | Jaime Altcheh |
| 010-0001-7 | CB C2 16017 | BO_P1 | POSITIVE | Male | 49 | BO | Bolivia; Cochabamba | Faustino Torrico |
| 010-0002-6 | CB C2 16032 | BO_E1 | POSITIVE | Female | 47 | BO | Bolivia; Cochabamba | Faustino Torrico |
| 010-0004-4 | CB C2 16028 | BO_E2 | POSITIVE | Male | 58 | BO | Bolivia; Cochabamba | Faustino Torrico |
| 010-0005-3 | CB C2 16055 | BO_E3 | POSITIVE | Female | 35 | BO | Bolivia; Cochabamba | Faustino Torrico |
| 010-0010-5 | CB C2 16092 | BO_P2 | POSITIVE | Male | 37 | BO | Bolivia; Cochabamba | Faustino Torrico |
| 010-0012-3 | CB C2 16147 | BO_P3 | POSITIVE | Female | 41 | BO | Bolivia; Cochabamba | Faustino Torrico |
| 010-0013-2 | CB C2 16101 | BO_N3 | NEGATIVE | Female | 44 | BO | Bolivia; Cochabamba | Faustino Torrico |
| 010-0014-1 | CB C2 16118 | BO_N1 | NEGATIVE | Male | 37 | BO | Bolivia; Cochabamba | Faustino Torrico |
| 010-0016-0 | CB C2 16033 | BO_N2 | NEGATIVE | Female | 56 | BO | Bolivia; Cochabamba | Faustino Torrico |
| 010-0017-9 | CB C2 16130 | BO_N5 | NEGATIVE | Female | 15 | BO | Bolivia; Cochabamba | Faustino Torrico |
| 010-0018-8 | CB C2 16122 | BO_N4 | NEGATIVE | Male | 38 | BO | Bolivia; Cochabamba | Faustino Torrico |
| 010-0020-2 | TJ C2 12818 | BO_P4 | POSITIVE | Female | 59 | BO | Bolivia; Tarija | Faustino Torrico |
| 010-0022-0 | TJ C2 12825 | BO_P5 | POSITIVE | Female | 27 | BO | Bolivia; Tarija | Faustino Torrico |
| 010-0024-9 | TJ C2 12828 | BO_E4 | POSITIVE | Female | 49 | BO | Bolivia; Tarija | Faustino Torrico |
| 010-0026-7 | TJ C2 12827 | BO_P6 | POSITIVE | Male | 50 | BO | Bolivia; Tarija | Faustino Torrico |
| 010-0027-6 | TJ C2 12844 | BO_E5 | POSITIVE | Male | 20 | BO | Bolivia; Tarija | Faustino Torrico |
| 010-0028-5 | TJ C2 12862 | BO_E6 | POSITIVE | Male | 54 | BO | Bolivia; Tarija | Faustino Torrico |
| 03-0000-8 | 0790001-005 | MX_N1 | NEGATIVE | Male | 52 | MX | Mexico | Janine Ramsey |
| 03-0001-7 | 0790001-009 | MX_N2 | NEGATIVE | Female | 38 | MX | Mexico | Janine Ramsey |
| 03-0002-6 | 5260001-009 | MX_P1 | POSITIVE | Female | 76 | MX | Mexico | Janine Ramsey |
| 03-0003-5 | 5260001-014 | MX_P2 | POSITIVE | Male | 79 | MX | Mexico | Janine Ramsey |
| 03-0004-4 | 5260001-016 | MX_N3 | NEGATIVE | Male | 46 | MX | Mexico | Janine Ramsey |
| 03-0005-3 | 5260047-001 | MX_P3 | POSITIVE | Female | 49 | MX | Mexico | Janine Ramsey |
| 03-0007-1 | 20380001-034 | MX_N4 | NEGATIVE | Female | 31 | MX | Mexico | Janine Ramsey |
| 03-0018-8 | 200790001-011 | MX_N5 | NEGATIVE | Male | 24 | MX | Mexico | Janine Ramsey |
| 03-0032-8 | 205260001-002 | MX_E1 | POSITIVE | Female | 96 | MX | Mexico | Janine Ramsey |
| 03-0033-7 | 205260001-003 | MX_E2 | POSITIVE | Female | 63 | MX | Mexico | Janine Ramsey |
| 03-0036-4 | 205260001-008 | MX_N6 | NEGATIVE | Female | 62 | MX | Mexico | Janine Ramsey |
| 03-0038-2 | 205260001-010 | MX_E3 | POSITIVE | Female | 65 | MX | Mexico | Janine Ramsey |
| 03-0046-1 | 205260018-001 | MX_P4 | POSITIVE | Female | 70 | MX | Mexico | Janine Ramsey |
| 03-0065-7 | 203800001-015 | MX_E4 | POSITIVE | Female | 48 | MX | Mexico | Janine Ramsey |
| 03-0067-5 | 203800001-017 | MX_E5 | POSITIVE | Female | 76 | MX | Mexico | Janine Ramsey |
| 03-0088-9 | 2052600080-001 | MX_P5 | POSITIVE | Female | 65 | MX | Mexico | Janine Ramsey |
| 03-0091-2 | 203800001-002 | MX_P6 | POSITIVE | Female | 47 | MX | Mexico | Janine Ramsey |
| 03-0094-0 | 203800001-005 | MX_E6 | POSITIVE | Male | 36 | MX | Mexico | Janine Ramsey |
| 05-0002-4 | 5951 | LE_P3 | NEGATIVE | Female | 10 | AR | Argentina | Jorge Diego Marco |
| 05-0003-3 | 5950 | LE_P2 | NEGATIVE | Female | 34 | AR | Argentina | Jorge Diego Marco |
| 05-0020-0 | 6112 | LE_N4 | NEGATIVE | Female | 28 | AR | Argentina | Jorge Diego Marco |
| 05-0023-8 | 5909 | LE_N2 | NEGATIVE | Male | 32 | AR | Argentina | Jorge Diego Marco |
| 05-0029-2 | 5530 | LE_N6 | NEGATIVE | Male | 23 | AR | Argentina | Jorge Diego Marco |
| 05-0036-2 | 5844 | LE_N1 | NEGATIVE | Female | 12 | AR | Argentina | Jorge Diego Marco |
| 05-0037-1 | 5921 | LE_N3 | NEGATIVE | Male | 14 | AR | Argentina | Jorge Diego Marco |
| 05-0038-0 | 6515 | LE_N5 | NEGATIVE | Male | 7 | AR | Argentina | Jorge Diego Marco |
| 05-0041-4 | 6110 | LE_P4 | NEGATIVE | Female | 68 | AR | Argentina | Jorge Diego Marco |
| 05-0043-2 | 6116 | LE_P6 | NEGATIVE | Male | 61 | AR | Argentina | Jorge Diego Marco |
| 05-0045-0 | 5954 | LE_P1 | NEGATIVE | Male | 8 | AR | Argentina | Jorge Diego Marco |
| 05-0046-0 | 6511 | LE_P5 | NEGATIVE | Male | 17 | AR | Argentina | Jorge Diego Marco |
| 07-0000-4 | Tcruzi-025 | US_P1 | POSITIVE | Male | 38 | US | USA | Melissa Nolan |
| 07-0001-3 | Tcruzi-032 | US_P2 | POSITIVE | Male | 58 | US | USA | Melissa Nolan |
| 07-0002-2 | Tcruzi-033 | US_P3 | POSITIVE | Female | 50 | US | USA | Melissa Nolan |
| 07-0003-1 | Tcruzi-044 | US_P4 | POSITIVE | Female | 63 | US | USA | Melissa Nolan |
| 07-0004-0 | Tcruzi-055 | US_P5 | POSITIVE | Male | 54 | US | USA | Melissa Nolan |
| 07-0005-0 | Tcruzi-056 | US_P6 | POSITIVE | Female | 27 | US | USA | Melissa Nolan |
| 07-0006-9 | H-153 | US_N1 | NEGATIVE | Male | 38 | US | USA | Melissa Nolan |
| 07-0007-8 | H-731 | US_N2 | NEGATIVE | Male | 58 | US | USA | Melissa Nolan |
| 07-0008-7 | H-714 | US_N3 | NEGATIVE | Female | 50 | US | USA | Melissa Nolan |
| 07-0009-6 | H-611 | US_N4 | NEGATIVE | Female | 63 | US | USA | Melissa Nolan |
| 07-0010-1 | H-052 | US_N5 | NEGATIVE | Male | 54 | US | USA | Melissa Nolan |
| 07-0011-0 | H-414 | US_N6 | NEGATIVE | Female | 27 | US | USA | Melissa Nolan |
| 07-0012-0 | Tcruzi-027 | US_E1 | POSITIVE | Male | 49 | US | USA | Melissa Nolan |
| 07-0013-9 | Tcruzi-046 | US_E2 | POSITIVE | Male | 73 | US | USA | Melissa Nolan |
| 07-0014-8 | Tcruzi-047 | US_E3 | POSITIVE | Male | 24 | US | USA | Melissa Nolan |
| 07-0015-7 | Tcruzi-059 | US_E4 | POSITIVE | Female | 52 | US | USA | Melissa Nolan |
| 07-0016-6 | Tcruzi-060 | US_E5 | POSITIVE | Female | 53 | US | USA | Melissa Nolan |
| 07-0017-5 | Tcruzi-061 | US_E6 | POSITIVE | Female | 42 | US | USA | Melissa Nolan |
| 08-0000-3 | 1065 | CO_P1 | POSITIVE | Female | 53 | CO | Santander; Colombia | Juan Carlos Villar |
| 08-0001-2 | 406 | CO_P2 | POSITIVE | Female | 52 | CO | Santander; Colombia | Juan Carlos Villar |
| 08-0002-1 | 43 | CO_P3 | POSITIVE | Male | 54 | CO | Santander; Colombia | Juan Carlos Villar |
| 08-0003-0 | 2230 | CO_P4 | POSITIVE | Male | 53 | CO | Santander; Colombia | Juan Carlos Villar |
| 08-0004-0 | 644 | CO_N1 | NEGATIVE | Female | 56 | CO | Santander; Colombia | Juan Carlos Villar |
| 08-0005-9 | 695 | CO_N2 | NEGATIVE | Female | 56 | CO | Santander; Colombia | Juan Carlos Villar |
| 08-0006-8 | 1639 | CO_N3 | NEGATIVE | Male | 56 | CO | Santander; Colombia | Juan Carlos Villar |
| 08-0007-7 | 1910 | CO_N4 | NEGATIVE | Male | 56 | CO | Santander; Colombia | Juan Carlos Villar |
| 08-0008-6 | 2002 | CO_E1 | POSITIVE | Female | 63 | CO | Santander; Colombia | Juan Carlos Villar |
| 08-0009-5 | 2100 | CO_E2 | POSITIVE | Female | 45 | CO | Santander; Colombia | Juan Carlos Villar |
| 08-0010-0 | 392 | CO_E3 | POSITIVE | Male | 48 | CO | Santander; Colombia | Juan Carlos Villar |
| 08-0011-0 | 2105 | CO_E4 | POSITIVE | Female | 48 | CO | Santander; Colombia | Juan Carlos Villar |
| 08-0012-9 | 2252 | CO_E5 | POSITIVE | Female | 57 | CO | Santander; Colombia | Juan Carlos Villar |
| 08-0013-8 | 2256 | CO_E6 | POSITIVE | Male | 56 | CO | Santander; Colombia | Juan Carlos Villar |
| 08-0014-7 | 2194 | CO_E7 | POSITIVE | Male | 35 | CO | Santander; Colombia | Juan Carlos Villar |
| 09-0000-2 | 1537 | BR_P1 | POSITIVE | Female | NA | BR | Brazil | Norival Kesper Jr |
| 09-0001-1 | 1539 | BR_P2 | POSITIVE | Female | NA | BR | Brazil | Norival Kesper Jr |
| 09-0003-0 | 1024 | BR_N1 | NEGATIVE | Female | NA | BR | Brazil | Norival Kesper Jr |
| 09-0004-9 | 847 | BR_N2 | NEGATIVE | Female | NA | BR | Brazil | Norival Kesper Jr |
| 09-0005-8 | 912 | BR_N3 | NEGATIVE | Female | NA | BR | Brazil | Norival Kesper Jr |
| 09-0006-7 | 1551 | BR_P3 | POSITIVE | Male | NA | BR | Brazil | Norival Kesper Jr |
| 09-0007-6 | 1534 | BR_P4 | POSITIVE | Male | NA | BR | Brazil | Norival Kesper Jr |
| 09-0008-5 | 1542 | BR_P5 | POSITIVE | Male | NA | BR | Brazil | Norival Kesper Jr |
| 09-0009-4 | 945 | BR_N4 | NEGATIVE | Male | NA | BR | Brazil | Norival Kesper Jr |
| 09-0010-0 | 984 | BR_N5 | NEGATIVE | Male | NA | BR | Brazil | Norival Kesper Jr |
| 09-0011-9 | 622 | BR_N6 | NEGATIVE | Male | NA | BR | Brazil | Norival Kesper Jr |
| 09-0012-8 | DP622 | BR_E1 | POSITIVE | NA | NA | BR | Brazil | Norival Kesper Jr |
| 09-0013-7 | DP630 | BR_E2 | POSITIVE | NA | NA | BR | Brazil | Norival Kesper Jr |
| 09-0014-6 | DP632 | BR_E3 | POSITIVE | NA | NA | BR | Brazil | Norival Kesper Jr |
| 09-0015-5 | DP633 | BR_E4 | POSITIVE | NA | NA | BR | Brazil | Norival Kesper Jr |
| 09-0016-4 | DP634 | BR_E5 | POSITIVE | NA | NA | BR | Brazil | Norival Kesper Jr |
| 09-0017-3 | DP635 | BR_E6 | POSITIVE | NA | NA | BR | Brazil | Norival Kesper Jr |
| 09-0021-6 | DP638 | BR_E7 | POSITIVE | NA | NA | BR | Brazil | Norival Kesper Jr |

*For 98 samples with available information, median=47.5; Mean=44.43.
