# Supplementary material for: Deep serological profiling of the Trypanosoma cruzi TSSA antigen reveals different epitopes and modes of recognition by Chagas disease patients
Source: PLoS Negl Trop Dis. 2023 Aug 9;17(8):e0011542. doi: 10.1371/journal.pntd.0011542 (PMC10441789; doi:10.1371/journal.pntd.0011542)
Supplement: S2 Table — (DOCX) [file pntd.0011542.s002.docx]

**Supplementary Table 2: Features of the TSSA peptide microarrays.**

| **Antigen** | **Sequence mapped** | **Peptide length** | **Serum sample** | **Number of peptides** | **Assay** |
| --- | --- | --- | --- | --- | --- |
| TSSAI | 1-91 | 15mer | Pool | 77 | Differential antibody recognition across endemic areas |
|  |  | 16mer | Individual | 76 |  |
| TSSAII | 1-92 | 15mer | Pool | 78 |  |
|  |  | 16mer | Individual | 77 |  |
| TSSAIII | 1-92 | 15mer | Pool | 78 |  |
|  |  | 16mer | Individual | 77 |  |
| TSSAIV | 1-92 | 15mer | Pool | 78 |  |
|  |  | 16mer | Individual | 77 |  |
| TSSAII | 24-62 | 13-8mer | Pool | 177 | Epitope fingerprinting |
| TSSA Chimera | 30-55 | 15mer | Pool | 3456 | TSSA diversity and antibody recognition |
| TSSAII | 31-50 | 16mer | Individual | 85 | Mutational scanning |
