# Supplementary material for: Deep serological profiling of the Trypanosoma cruzi TSSA antigen reveals different epitopes and modes of recognition by Chagas disease patients
Source: PLoS Negl Trop Dis. 2023 Aug 9;17(8):e0011542. doi: 10.1371/journal.pntd.0011542 (PMC10441789; doi:10.1371/journal.pntd.0011542)
Supplement: S3 Table — (DOCX) [file pntd.0011542.s003.docx]

**Supplementary Table 3: Features of TSSA sequences in Trypanosomatids.**

| **Sequence ID** | **Strain/Clone** | **DTU** | **TSSA isoform** | **Copy**  **number^1^** | **Identical to** | **Database** |
| --- | --- | --- | --- | --- | --- | --- |
| WWPY01000272.1 | *B. M. Lopez* | TcI | I | 1 | TcBrA4_Chr5 | NCBI |
| TcBrA4_Chr5 | *Brazil A4* | TcI | I | 7 \| 2 | **TSSAI var. 3^2^** | TriTryp |
| PYLG01001191.1 | *Colombiana* | TcI | I | 1 | PRFA01000186 | NCBI |
| AYLP01000047 | *Dm28c 2014* | TcI | I | 1 | PRFA01000186 | NCBI |
| MBSY01000062 | *Dm28c 2017* | TcI | I | 3 | PRFA01000017 | NCBI |
| MBSY01000671 | *Dm28c 2017* | TcI | I | 14 \| 5 | PRFA01000186 | NCBI |
| PRFA01000017 | *Dm28c 2018* | TcI | I | 3 | **TSSAI var. 2** | NCBI |
| PRFA01000186 | *Dm28c 2018* | TcI | I | 8 | **TSSAI var. 4** | NCBI |
| MKKV01000002.1 | *T. cruzi G* | TcI | I | 1 | PRFA01000017 | NCBI |
| KB222892.1 | *T. cruzi JR cl. 4* | TcI | I | 1 | PRFA01000186 | TriTryp |
| TcX10_chr27 | *Sylvio X10/1* | TcI | I | 7 \| 5 | **TSSAI Ref. variant** **(AFF60282.1)** | TriTryp |
| ADWP02013979 | *Sylvio X10/1-2012* | TcI | I | 1 | AFF60282.1 | NCBI |
| JABDHM010000021.1 | *Berenice* | TcII | II | 13^3^ | TcCLB.507511.91 | NCBI |
| JABDHM010000549.1 | *Berenice* | TcII | II | 5 \| 1 | TcCLB.507511.91 | NCBI |
| JABDHM010000888.1 | *Berenice* | TcII | II | 1 | TcCLB.507511.91 | NCBI |
| PYKV01000362.1 | *11* | TcII | II | 1 | PYKZ01000655.1 | NCBI |
| PYLE01001059.1 | *S15* | TcII | II | 1 | TcCLB.507511.91 | NCBI |
| PYLB01000089.1 | *S23b* | TcII | II | 1 | TcCLB.507511.91 | NCBI |
| PYLA01000455.1 | *S44a* | TcII | II | 1 | TcCLB.507511.91 | NCBI |
| PYKZ01000655.1 | *S92a* | TcII | II | 1 | **TSSAII var. 2** | NCBI |
| ANOX01005286.1 | *Esmeraldo* | TcII | II | 1 | **TSSAII var. 3** | NCBI |
| tig00001302 | *Y* | TcII | II | 9 | TcCLB.507511.91 | This work |
| TcYC6_Chr11 | *Y C6* | TcII | II | 4 | TcCLB.507511.91 | TriTryp |
| PYKY01000419.1 | *Ycl2* | TcII | II | 1 | TcCLB.507511.91 | NCBI |
| PYKX01000041.1 | *Ycl4* | TcII | II | 1 | TcCLB.507511.91 | NCBI |
| NMZO01000011.1 | *Y A4* | TcII | I | 1 | AFF60282.1^4^ | NCBI |
| WWPZ01001089.1 | *Ikiakarora* | TcIII | II | 1 | **TSSAII var. 4**^4^ | NCBI |
| OGCJ01000576.1 | *231* | TcIII | III | 1 | TcCLB.508235.20 | NCBI |
| tig00000697 | *MT3663* | TcIII | III | 5 | **TSSAIII var. 2** | This work |
| tig00000211 | *Jose Julio* | TcIV | IV | 5 | **TSSAIV var. 2** | This work |
| tig00005616 | *BOL-FC10A* | TcV | II | 15 \| 3 | TcCLB.507511.91 | This work |
| tig00000089 | *BOL-FC10A* | TcV | III | 2 \| 1 | JACCJE010000048.1 | This work |
| NMZN01000018.1 | *Bug2148* | TcV | I | 5 | AFF60282.1^4^ | NCBI |
| NMZN01000356.1 | *Bug2148* | TcV | I | 1 | AFF60282.1^4^ | NCBI |
| JACCJE010000048.1 | *SC43* | TcV | II | 12 \| 1 | TcCLB.507511.91 | NCBI |
| JACCJE010000157.1 | *SC43* | TcV | III | 1 | **TSSAIII var. 3** | NCBI |
| MKQG01000016.1 | *CL* | TcVI | II | 1 | TcCLB.507511.91 | NCBI |
| TcChr35-S | *CL Brener Esmeraldo-like* | TcVI | II | 2 | **TSSAII Ref. variant (TcCLB.507511.91**) | TriTryp |
| 341_RA | *RA* | TcVI | II | 12 \| 2 | TcCLB.507511.91 | This work |
| PRFC01000055 (TCC55) | *TCC* | TcVI | II | 12 \| 1 | TcCLB.507511.91 | NCBI |
| KB851849.1 | *Tula cl2* | TcVI | II | 1 | TcCLB.507511.91 | TriTryp |
| TcChr37-P | *CL Brener Non-Esmeraldo-like* | TcVI | III | 1 | **TSSAIII Ref. variant (TcCLB.508235.20)** | TriTryp |
| 2244_RA | *RA* | TcVI | III | 1 | TcCLB.508235.20 | This work |
| PRFC01000216 (TCC216) | *TCC* | TcVI | III | 1 | TcCLB.508235.20 | NCBI |
| KB851474.1 | *Tula cl2* | TcVI | III | 1 | TcCLB.508235.20 | TriTryp |
| TcMARK_CONTIG_1819 | *Marinkellei strain B7* |  |  | 1 | **TcMARK TSSA** | TriTryp |

^1^ Copy number refers to the total number of TSSA-related sequences and is expressed as number of coding genes | number of pseudogenes.

^2^ The different variants for each isoform are highlighted in bold.

^3^ Synonymous polymorphisms were detected.

^4^ Discordance between informed DTU and TSSA isoform.
