# Supplementary material for: Deep serological profiling of the Trypanosoma cruzi TSSA antigen reveals different epitopes and modes of recognition by Chagas disease patients
Source: PLoS Negl Trop Dis. 2023 Aug 9;17(8):e0011542. doi: 10.1371/journal.pntd.0011542 (PMC10441789; doi:10.1371/journal.pntd.0011542)
Supplement: S4 Table — (DOCX) [file pntd.0011542.s004.docx]

**Supplementary Table 4: Sequences of previously undescribed TSSA genes.**

| **Sequence ID** | **DNA sequence** | **Translated sequence** |
| --- | --- | --- |
| tig00001302 | ATGACTACGTGCCGTCTGCTGTGCGCCCTGTTGGCGCTTGCCCTGTGCTGCTGCCTAACTGCGTGCACGACAGCGAATGGTGGGTCTACTAGTTCTACCCCACCTTCTGGTACGGAAAATAAACCAGCTACAGGGGAAGCTCCATCTCAACCGGGGGCTTCTTCAGGTGAAGCAGAAGCCTCCTCAAATAAGAATGACGGCAGCCTCAGCAGCTCTGCGTGGGTGAGTGCCCCGCTGGCGCTCGCCGCATCCGCGCTGGCGTACACCGCTCTGGGCTGA | MTTCRLLCALLALALCCCLTACTTANGGSTSSTPPSGTENKPATGEAPSQPGASSGEAEASSNKNDGSLSSSAWVSAPLALAASALAYTALG* |
| tig00000697 | ATGGCTACGTGCCGTCTGCTGTGCGCCCTGTTGGCGCTTGCCCTGTGCTGCTGCCTATCTGCGTGCACGACAGCGAATGGTGGGTCTACTATTTCTACCCCACCTTCTGGTGCGGACAAGAAAACAGCTGCAGGGGAAGCTCCATCTCCATCGGGAGCTTCTTCAGGTGAAGCAGAAGCCTCCTCAAATAAGAATGACGGCAGCCTCAGCAGCTCTGCGTGGGCGTTTGCCCCGCTGGCGCTCGCCGCATCCGCGCTGGCGTACACCGCTCTGGGCTGA | MATCRLLCALLALALCCCLSACTTANGGSTISTPPSGADKKTAAGEAPSPSGASSGEAEASSNKNDGSLSSSAWAFAPLALAASALAYTALG* |
| tig00000211 | ATGGCTACGTGCCGTCTGCTGTGCGCCCTGTTGGCGCTTGCCCTGTGCTGCTGCCTATCTGCGTGCACGACAGCGAATGGTGGGTCTACTATTTCTACCCCACCTTCTGGTGCGGACAAGAAAACAGCTGCAGGGGAAGCTCCATCTCCATCGGGAGCTTCTTCAGGTGAAGCAGAAGCCTCCTCAAATAAGAATGACGGCAGCCTCAGCAGCTCTGCGTGGGCGTTTGCCCCGCTGGCGCTCGCCGCATCCGCGCTGGCGTACACCGCTCTGGGCTGA | MATCRLLCALLALALCCCLSACTTANGGSTISTPPSGADKKTAAGEAPSPSGASSGEAEASSNKNDGSLSSSAWAFAPLALAASALAYTALG* |
| tig00005616 | ATGACTACGTGCCGTCTGCTGTGCGCCCTGTTGGCGCTTGCCCTGTGCTGCTGCCTAACTGCGTGCACGACAGCGAATGGTGGGTCTACTAGTTCTACCCCACCTTCTGGTACGGAAAATAAACCAGCTACAGGGGAAGCTCCATCTCAACCGGGGGCTTCTTCAGGTGAAGCAGAAGCCTCCTCAAATAAGAATGACGGCAGCCTCAGCAGCTCTGCGTGGGTGAGTGCCCCGCTGGCGCTCGCCGCATCCGCGCTGGCGTACACCGCTCTGGGCTGA | MTTCRLLCALLALALCCCLTACTTANGGSTSSTPPSGTENKPATGEAPSQPGASSGEAEASSNKNDGSLSSSAWVSAPLALAASALAYTALG* |
| tig00000089 | ATGGCTACGTGCCGTCTGCTGTGCGCCCTGTTGGCGCTTGCCCTGTGTTGCTGCCTATCTGCGTGCACGACAGCGAATGGTGGGTCTACTATTTCTACCCCACCTTCTGGTACGGAGAAGAAAGCAGCTGCAGGGGAAGCTCCATCTCCATCGGGAGCTTCTTCAGGTGAAGCAGAAGCCTCCTCAAATAAGAATGACGGCAGCCTCAGCAGCTCTGCGTGGGTGTTTGCCCCGCTGGCGCTCACCGCATCCGCGCTGGCGTACACCGCTCTGAGCTGA | MATCRLLCALLALALCCCLSACTTANGGSTISTPPSGTEKKAAAGEAPSPSGASSGEAEASSNKNDGSLSSSAWVFAPLALTASALAYTALG* |
| 341_RA | ATGACTACGTGCCGTCTGCTGTGCGCCCTGTTGGCGCTTGCCCTGTGCTGCTGCCTAACTGCGTGCACGACAGCGAATGGTGGGTCTACTAGTTCTACCCCACCTTCTGGTACGGAAAATAAACCAGCTACAGGGGAAGCTCCATCTCAACCGGGGGCTTCTTCAGGTGAAGCAGAAGCCTCCTCAAATAAGAATGACGGCAGCCTCAGCAGCTCTGCGTGGGTGAGTGCCCCGCTGGCGCTCGCCGCATCCGCGCTGGCGTACACCGCTCTGGGCTGA | MTTCRLLCALLALALCCCLTACTTANGGSTSSTPPSGTENKPATGEAPSQPGASSGEAEASSNKNDGSLSSSAWVSAPLALAASALAYTALG* |
| 2244_RA | ATGGCTACGTGCCGTCTGCTGTGCGCCCTGTTGGCGCTTGCCCTGTGTTGCTGCCTATCTGCGTGCACGACAGCGAATGGTGGGTCTACTATTTCTACCCCACCTTCTGGTACGGAGAAGAAAGCAGCTGCAGGGGAAGCTCCATCTCCATCGGGAGCTTCTTCAGGTGAAGCAGAAGCCTCCTCAAATAAGAATGACGGCAGCCTCAGCAGCTCTGCGTGGGTGTTTGCCCCGCTGGCGCTCACCGCATCCGCGCTGGCGTACACCGCTCTGGGCTGA | MATCRLLCALLALALCCCLSACTTANGGSTISTPPSGTEKKAAAGEAPSPSGASSGEAEASSNKNDGSLSSSAWVFAPLALTASALAYTALG* |
