# Supplementary figures and images for: Deep serological profiling of the Trypanosoma cruzi TSSA antigen reveals different epitopes and modes of recognition by Chagas disease patients
Source: PLoS Negl Trop Dis. 2023 Aug 9;17(8):e0011542. doi: 10.1371/journal.pntd.0011542 (PMC10441789; doi:10.1371/journal.pntd.0011542)

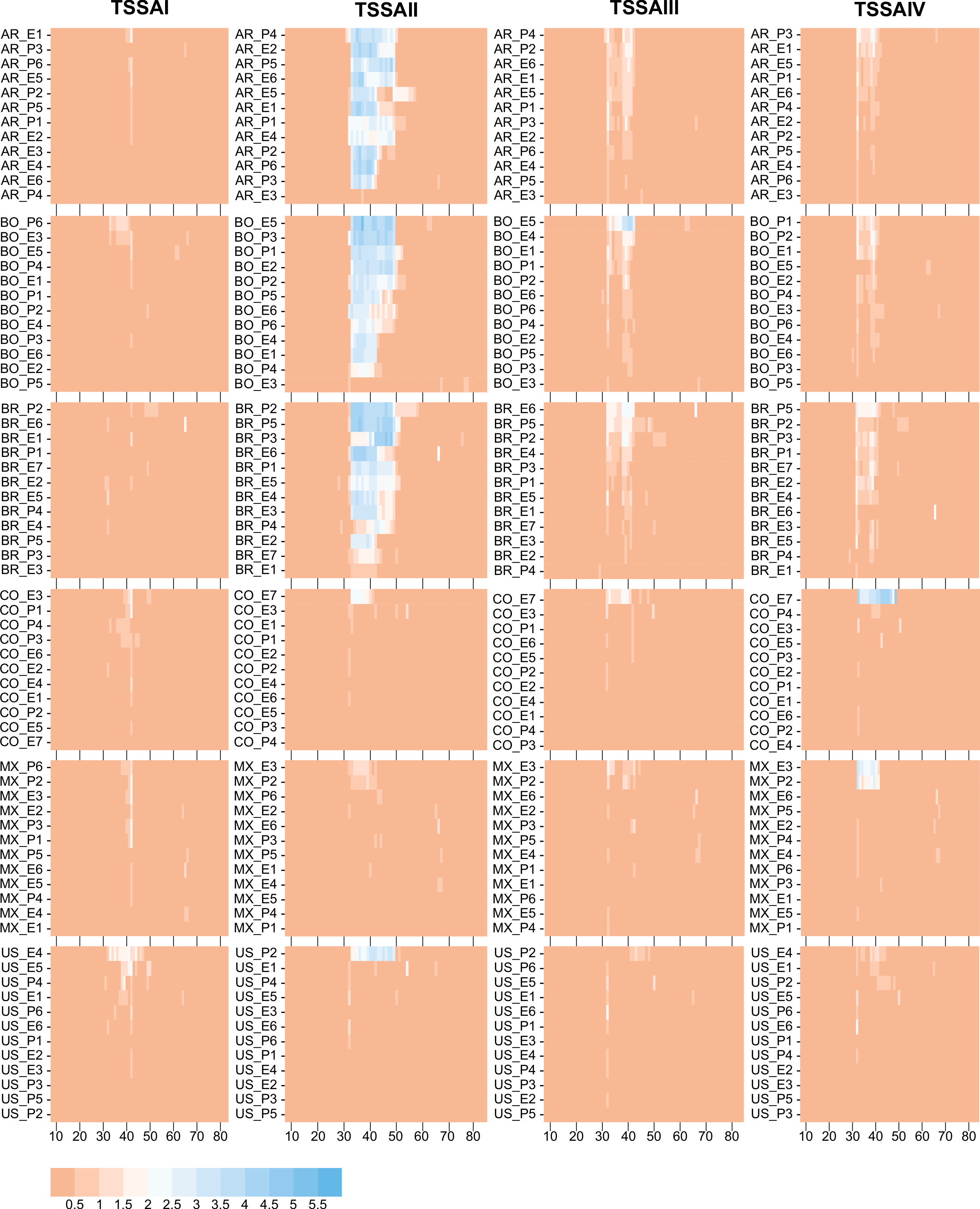

Supplement: S1 Fig — Peptide arrays comprising sets of fully overlapped 16mers and encompassing the complete sequence of TSSAI, TSSAII, TSSAIII or TSSAIV reference variants were probed with 71 serum samples from chronic Chagas disease patients of different geographic origin (‘AR’ = Argentina; ‘BO’ = Bolivia; ‘BR’ = Brazil; ‘CO’ = Colombia; ‘MX’ = Mexico; ‘US’ = United States). The mean reactivity of each residue in the context of individual sequences is indicated with a color scale. For each TSSA isoform, samples from each geographic origin were ordered according to their reactivity. (TIFF) [file pntd.0011542.s006.tiff]

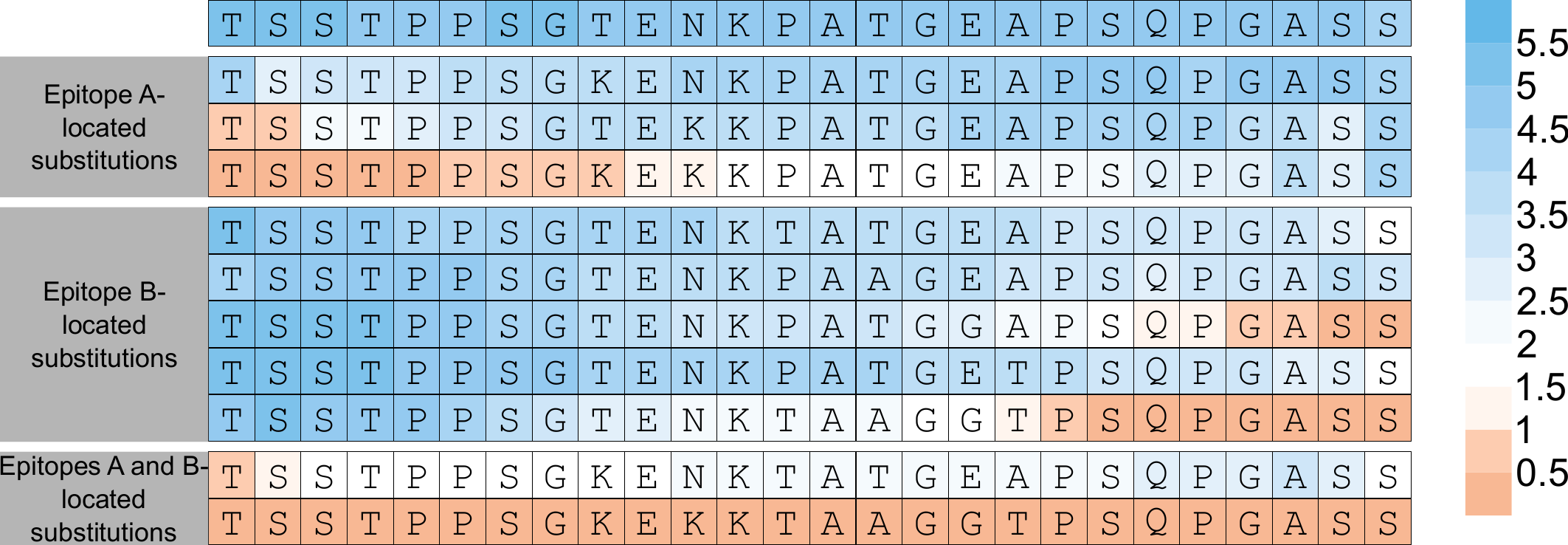

Supplement: S2 Fig — Peptide arrays comprising sets of fully overlapped 15mers, encompassing residues 30 to 55 of TSSAII reference variant (TSSAII30-55) and bearing selected substitution(s) at polymorphic positions were probed with a pool of sera of chronic Chagas disease patients from Bolivia. The mean reactivity of each residue in the context of wild-type (top row) and single- or multiple-substituted sequences is indicated with a color scale. For each sequence, the replaced residue(s) is/are indicated. (TIFF) [file pntd.0011542.s007.tiff]

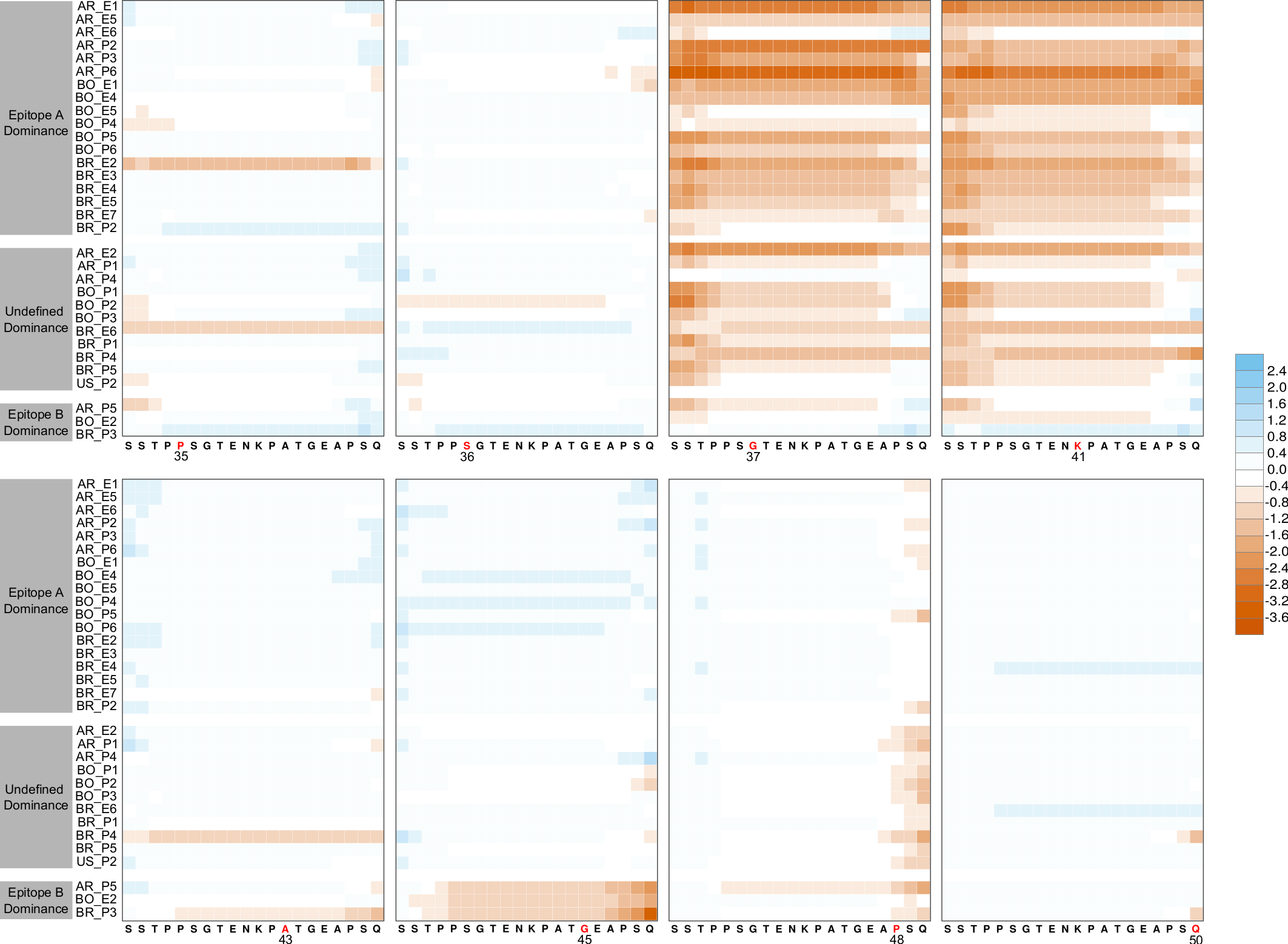

Supplement: S3 Fig — Peptide arrays comprising sets of fully overlapped 16mers, encompassing residues 31 to 50 of TSSAII reference variant (TSSAII31-50) were probed with chronic Chagas disease sera from the indicated geographical origin. The net impact caused by selected Alanine substitutions (indicated in red) was calculated as the difference in reactivity for each residue between the original and mutated sequences. This difference in reactivity is indicated with a color scale. In the case of Alanine 43 (bottom left panel), it was mutated to G. (TIFF) [file pntd.0011542.s008.tiff]

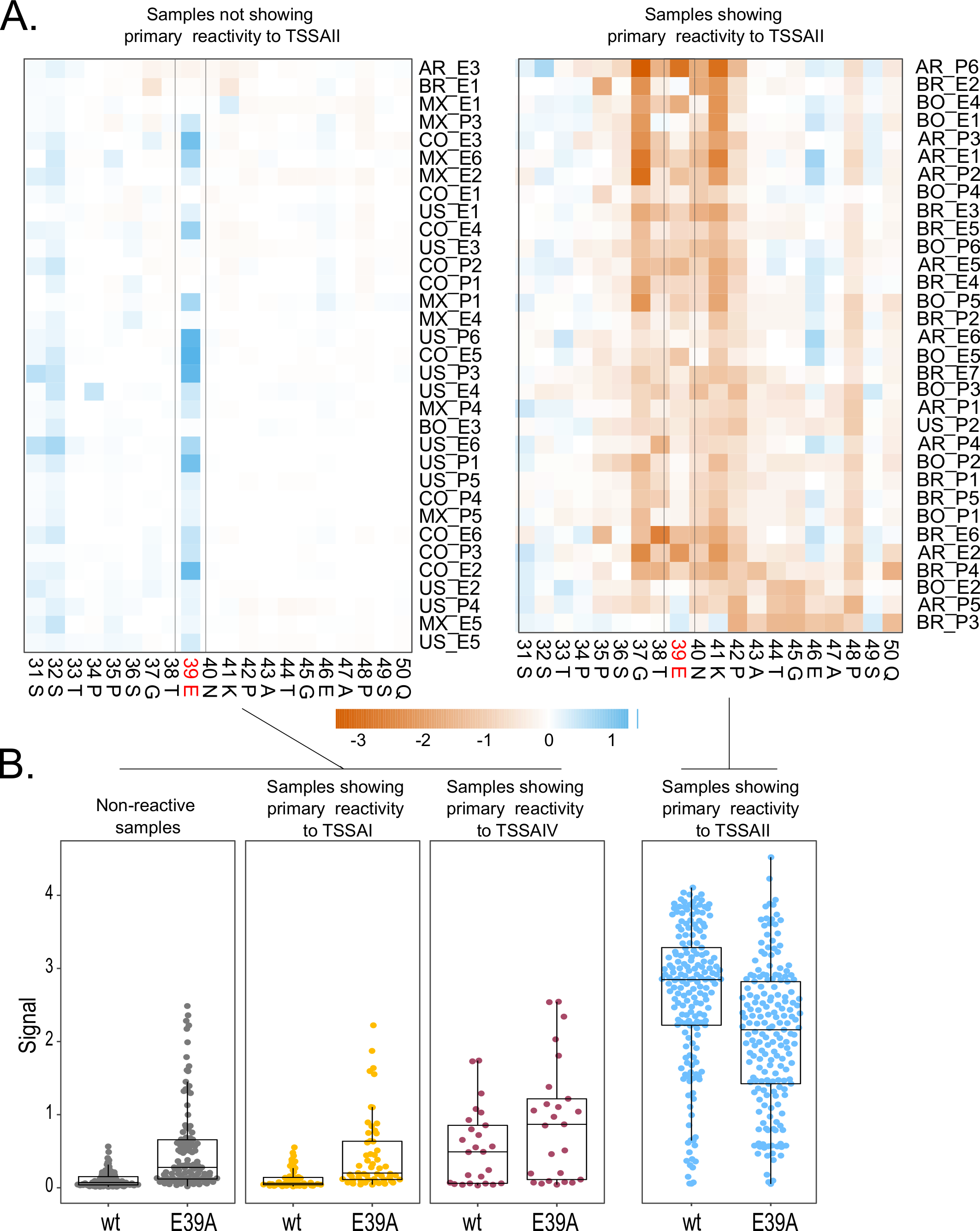

Supplement: S4 Fig — A) Heatmap showing the overall impact of a specific replacement on TSSAII31-50 recognition by Chagas disease samples either showing (right panel, n = 32) or not showing (left panel, n = 33) primary reactivity to TSSAII (for further details see legend to Fig 6). In both panels, the line corresponding to the E39A replacement is highlighted. B) Impact of E39A substitution on the reactivity to TSSAII31-50 of TSSA-negative serum samples (left panel) or from serum samples showing primary reactivity to TSSAI, TSSAIV or TSSAII. The reactivity of all peptides containing the E39A substitution is shown for each serum sample (in each case, the median ± SD are indicated by box and whiskers). (TIFF) [file pntd.0011542.s009.tiff]
