# Supplementary material for: Deep serological profiling of the Trypanosoma cruzi TSSA antigen reveals different epitopes and modes of recognition by Chagas disease patients
Source: PLoS Negl Trop Dis. 2023 Aug 9;17(8):e0011542. doi: 10.1371/journal.pntd.0011542 (PMC10441789; doi:10.1371/journal.pntd.0011542)
Supplement: Attachment — Submitted filename: Response to reviewers.docx [file pntd.0011542.s013.docx]

**Responses to Reviewers**

***Reviewer #1:*** This is a detailed analysis of the Trypanosoma cruzi antigen TSSA, revealing new diversity, with relevance to geographical distribution and to diagnostic sensitivity. This rigorous research will contribute to understanding of the epidemiology of Chagas disease, and to improvement of regional diagnostic efficacy. The methods are complex, and results detailed. However, in the Discussion, some of the results are already described or are expected, for example, as with samples from TSSA1 and TSSAIV, for which it is well-known where these infections predominate. The two variants of TSSA1 are interesting and their epidemiology worthy of follow up, with wider comparative genomics.
There are no significant flaws, and the manuscript is worthy of publication.
A few minor adjustments are recommended, as follows:
Line 174-175. The authors should clarify in the legend whether the letters ‘A’, ‘C’ , ‘D’ etc, in Figure 1B are or are not the standard single-letter abbreviations for amino acids (as used in Figure 1A).

**Yes, they are. This has been amended in the legend to Figure 1B.**

Line 227. The authors should state (n = 30), rather than simply ‘{30}’.

**Here the ‘(30)’ refers to the reference #30 (Durante IM, La Spina PE, Carmona SJ, Agüero F, Buscaglia CA. High-resolution profiling of linear B-cell epitopes from mucin-associated surface proteins (MASPs) of Trypanosoma cruzi during human infections. PLoS Negl 812 Trop Dis. 2017;11), not to the number of strains of T. cruzi.**

Line 282. Replace the word ‘above’ with ‘top’.

**Corrected.**

Line 515-516. Do the authors mean ‘Table 1’ here in the text (not 'Table 2')?

**Corrected.**

Table 1. In the column headings, the authors should add the superscript numbers to the TSSA peptides as done in the text, for example TSSAIV 24-62

**Corrected.**

***Reviewer #2:*** The authors have a long history in exploring the serological response to the TSSA antigen for the identification of T. cruzi DTU infecting strain.
The manuscript describes a thoughtful work to characterize the linear B-cell epitope profiling of TSSA and reveals variations in the seroprevalence and on the specificity of TSSA antibodies among Chagas disease populations. The findings show a good correlation between the identified TSSA isoform(s) and the reported DTU.

Major points
1. Authors’ citations
- In the manuscript, extensive self-citation of co-authors is noted. Important references are not cited. I invite authors to revise the recent literature. For example, the review by Magalhães et al. 2022 (Lancet Microbe doi: 10.1016/S2666-5247(21)00265-2) on T. cruzi pathogen diversity, immunity, and the fate of infections should clearly be mentioned in the Introduction and Discussion.

**This reference has been added**

- Introduction
Line 103.  methods able to reliably assign the infecting strain
Authors should mention that molecular methods are available to genotype the infecting strain DTU and their limitations (see Zingales et al. 2012 MEEGID doi:10.1016/j.meegid.2011.12.009).

**We appreciate the reviewer’s comment. However, the advantages and limitations of currently available genotyping methods have been extensively discussed and were thereby not mentioned here. We rephrased this by stating ‘methods able to reliably assign the infecting strain type directly from biological samples are expected to have a positive effect on epidemiologic surveillance, prevention, and clinical management of Chagas disease’.**

- Discussion
Line 530. “as distinct DTUs seem to be associated with differential susceptibility to drugs, disease features and infection outcomes [40]. Reference [40] does not illustrate all the information in the sentence. To this reference must be added [refs 6, 7] and
Zingales 2018. Acta Tropica [dx.doi.org/10.1016/j.actatropica.2017.09.017](http://dx.doi.org/10.1016/j.actatropica.2017.09.017)
Magalhães et al., 2022. Lancet Microbe doi: 10.1016/S2666-5247(21)00265-2.

**These references were included**

- Reference citation.
The citation of references in the text must be standardized. Sometimes references are cited between square brackets, sometimes between parentheses, sometimes as a superscript.

**We apologize for this; the citation of references has been now standardized.**

2. The sera used are a fundamental element of the study. The authors must justify the choice and characterize the patients/sera.
- Justify the six countries whose sera were analyzed.
In the Abstract and in Line 139, it is mentioned “From different endemic settings” and "from different endemic areas". Regions of the USA cannot be considered “endemic”.
Please revise.

**We aimed to get a deep serological profiling of TSSA reactivity, and that’s why we analyzed sera from different settings across the Americas. As per the second part of the comment, this statement has been corrected. However, it should be noted that USA based patients are uniquely verified locally acquired cases as noted in the following articles:** [**https://pubmed.ncbi.nlm.nih.gov/28221110/**](https://pubmed.ncbi.nlm.nih.gov/28221110/) **and** [**https://pubmed.ncbi.nlm.nih.gov/25371187/**](https://pubmed.ncbi.nlm.nih.gov/25371187/)**. While the geographically southern USA is not hyperendemic for locally acquired transmission, the scientific literature has documented established triatomine and sylvatic transmission dating back to the early 1850s and the sustained rare occurrence of human transmission in focal areas. Therefore, the ability to incorporate the cases adds a unique value.**

- The high number of human sera from the USA (n=18) draws attention.
Most likely the patients are migrants from Latin American countries. This aspect should be mentioned in the article.

**This statement has been added. As for the number of US sera, it is 12, not 18. The same number of serum samples (n=12) were analyzed from each setting, except for Colombia that were 11.**

- In M&M, Page 6. Line 149, the authors state “The complete information of the population study can be found in [28]”. This reference needs to be updated. Ricci et al. 2023 Nature Communications | (2023) 14:1850.

**Done.**

On the other hand, it is important that the present article reports for EACH COUNTRY the number of the analyzed sera as well as the gender distribution and the mean and standard deviation of age of the patients. It is known that the immune response to T. cruzi varies with these parameters. Please note that for the sera from Argentina the gender of the patients is not specified in Suppl Data 2 by Ricci et al. The sera were provided by Dr. Altcheh, co-author of the present manuscript.

**The number of analyzed sera for each country has been included. We are now also providing an additional Supplementary Table (S1 Table) containing a complete list of all sera analyzed, for each country, as well as the sex and age where this information was available. Also, please note that we cannot provide information on gender because this is a socially-constructed role (See** [**https://doi.org/10.1186/s41073-016-0007-6**](https://doi.org/10.1186/s41073-016-0007-6)**). We can only report sex instead, and only where this information was available.** 

- The authors claim that the patients were asymptomatic (indeterminate ChD form). What tests were performed for this diagnosis? Why is it important for the study that the patients are asymptomatic? Please, inform this in the article.
**Analyzed patients displayed normal ECG values and results in routine clinical analysis (Balouz et al. 2017 Journal of Clinical Microbiology). We focused on this population to avoid possible serological bias due to *T. cruzi* associated pathologies.**

***Reviewer #3:*** In the present study, Romer et al. performed a deep linear B cell epitope mapping on TSSA (trypomastigote small surface antigen), as adhesin the elicit a strong antibody response during T. cruzi infections. Sera from Chagas disease patients from distinct endemic settings in the Americas, where different parasite DTUs circulate, were used to comprehensively evaluate the reactivity of peptides derived from TSSA sequences from different parasite isolates available in public databases. TSSA isoforms displayed different seroprevalence among Chagas disease patients correlating with differential distribution of parasite DTUs in the Americas. Ala-scan approach allow the identification of critical residues involved in the antibody binding. New TSSA variants and epitopes were identified, including a T. cruzi marinkellei sequence that reacted with serum from a Chagas disease patient from Colombia. This paper represents an important contribution for the study of TSSA linear
B-cell epitopes and deserves publication after some aspects are clarified.

Lines 146-149:
Sample naming: What is the letter “E” in the suffix?

**Samples with a letter 'P' in the suffix are samples that were part of the corresponding sample pools analyzed as part of the antigen and epitope discovery screening (CHAGASTOPE-v1 arrays in Ricci et al 2023). Samples with a letter 'E' in the suffix are samples that were not part of this discovery screening and were only analyzed on the CHAGASTOPE-v2 array designs. As explained in the paper this is the array design that contains all TSSA variants, as well as the peptide variants for the Alanine-scanning experiments. A statement explaining these details has been added in the Methods section.**

Please, provide more information regarding the microarray design, including the complete list of peptides that were evaluated in this study and the raw fluorescence values for each assayed peptide in each experiment.

**Agreed. We apologize for the omission of this information in the original submission. We are now including more information as required. Further details of peptide microarrays used in this work can be found in the S2 Table and at the ArrayExpress database Functional Genomics Data Collection (**[**https://www.ebi.ac.uk/biostudies/arrayexpress/arrays/**](https://www.ebi.ac.uk/biostudies/arrayexpress/arrays/)**) under accession codes A-MTAB-692 and A-MTAB-693 (Array Designs); and E-MTAB-11651 and E-MTAB-11655 (Assay Data).** **We are also providing now a supplementary file (S1 file) containing the sequences and raw fluorescence values for the peptides that were evaluated in this study. S1 file and the R scripts can be found at** [**https://github.com/trypanosomatics/The-Chagas-Disease-Antigen-and-Epitope-Atlas/tree/main/77_romer_et_al_TSSA.**](https://github.com/trypanosomatics/The-Chagas-Disease-Antigen-and-Epitope-Atlas/tree/main/77_romer_et_al_TSSA.)

Provide the public accession numbers for all the TSSA reference sequences (Figure 1).

**Public accession numbers for TSSA reference sequences have been added to the legend of Figure 1**

Legend figure 2: Please state what is the panel “isoforms recognized”

**We apologize, this was indeed confusing. We added the following statement to Figure 2 legend to clarify this issue: ’In every case, the overall TSSA seroprevalence is indicated by pie charts to the left. The bar charts show the primary reactivity (the most reactive TSSA isoform, left chart), and the number of isoforms recognized by TSSA-reactive sera (right chart). The y-axis refers to the number of samples’.**

Specify the genome versions as well as the public accession numbers included in the phylogenetic analysis. For genomes not publicly available, please provide the accession number of each gene/protein sequence used in this study or provide all sequences as supplementary material data.

**Genome versions and public accession numbers for each sequence are indicated in Suppl Table 3. Sequences retrieved from genomes not publicly available are provided in Suppl Table 4.**

Please provide the nucleotide sequence of the constructs of the (GST)-fusion proteins as supplemental material.
**Sequences of (GST)-fusion proteins are provided in Suppl Table 5.**

Please include in the Table S2 the database from which each sequence was retrieved.
**Done. Please note that original table S2 has been renamed as S3 table.**

Please describe the GST-Ag1 (control) used in the Table 1.

**This has been described in ref Ibañez et al, MBP 1987 (now included within the references)**


Typo:
Line 64: Trypanosoma cruzi: italic

**Done**
