# Supplementary material for: Deep serological profiling of the Trypanosoma cruzi TSSA antigen reveals different epitopes and modes of recognition by Chagas disease patients
Source: PLoS Negl Trop Dis. 2023 Aug 9;17(8):e0011542. doi: 10.1371/journal.pntd.0011542 (PMC10441789; doi:10.1371/journal.pntd.0011542)
Supplement: Attachment — Submitted filename: Response to reviewers.docx [file pntd.0011542.s014.docx]

**Responses to Reviewers**


***Reviewer #3:***

Two points are still pending:

1- The accession numbers of the Figure 1 sequences are still missing (they are not in the legend of Figure 1).

**This has been addressed**

2- Please provide the complete list of TSSA peptide sequences and their raw fluorescence values evaluated in this study as a supplementary file that will be hosted in the PNTD website (and not in the github author’s account).

**The complete list of TSSA peptide sequences evaluated in this study and their raw fluorescence values were included as supplementary files 1, 2 and 3.**

**Supplementary file 1 (**[**00_TSSA_variants_len16_allSingles_noReplicas.tsv**](https://drive.google.com/file/d/17BQEBLb-aJejRz6heVeBTkI3hP-wWa_e/view?usp=sharing)**): Complete list and reactivities of TSSA 16mer peptide sequences evaluated with individual serum samples and referred to in Fig. 2.**

**Supplementary file 2 ([TSSA_variants_all_pools_averagedReplicas.tsv](https://drive.google.com/file/d/1Y5dUT4XFLYpu11BBERm3sDePY0I_mFU9/view?usp=sharing)): Complete list and reactivities of TSSA sequences of variable length and chimeric sequence analyzed with pools of sera and referred to in Figs. 3 and 5.**

**Supplementary file 3 (**[**00_TSSA_variants_len16_ALASCAN_allSingles_noReplicas**](https://drive.google.com/file/d/1cXELfIYf8QyiY89HHlFVaobtpPlLMRZ1/view?usp=drive_link)**): Complete list and reactivities of TSSA sequences used for the mutational scanning and referred to in Fig. 6.**
